# Significance of Temporal Muscle Thickness in Chronic Subdural Hematoma

**DOI:** 10.3390/jcm11216456

**Published:** 2022-10-31

**Authors:** Daniel Dubinski, Sae-Yeon Won, Bedjan Behmanesh, Daniel Cantré, Isabell Mattes, Svorad Trnovec, Peter Baumgarten, Patrick Schuss, Thomas M. Freiman, Florian Gessler

**Affiliations:** 1 Department of Neurosurgery, Rostock University Medical Center, 18057 Rostock, Germany; 2 Institute of Diagnostic and Interventional Radiology, Pediatric Radiology and Neuroradiology, Rostock University Medical Center, 18057 Rostock, Germany; 3 Department of Neurosurgery, University Hospital, Schiller University Jena, 07747 Jena, Germany; 4 Department of Neurosurgery, Unfallkrankenhaus Berlin, 12683 Berlin, Germany

**Keywords:** temporal muscle thickness, sarcopenia, chronic subdural hematoma, recurrence, outcome, risk factors

## Abstract

Background: Reduced temporal muscle thickness (TMT) was verified as an independent negative prognostic parameter for outcome in brain tumor patients. Independent thereof, chronic subdural hematoma (CSDH) is a neurosurgical condition with high recurrence rates and unreliable risk models for poor outcome. Since sarcopenia was associated with poor outcome, we investigated the possible role of TMT and the clinical course of CSDH patients. Methods: This investigation is a single-center retrospective study on patients with CSDH. We analyzed the radiological and clinical data sets of 171 patients with surgically treated CSDH at a University Hospital from 2017 to 2020. Results: Our analysis showed a significant association between low-volume TMT and increased hematoma volume (*p* < 0.001), poor outcome at discharge (*p* < 0.001), and reduced performance status at 3 months (*p* < 0.002). Conclusion: TMT may represent an objective prognostic parameter and assist the identification of vulnerable CSDH patients.

## 1. Introduction

Chronic subdural hematoma (CSDH) is the most common neurosurgical condition, and with the increasing age of the Western population, the incidence of CSDH is expected to further increase in the near future [1]. To date, the major challenges in the management of CSDH is the difficult prognostication of outcome and high recurrence with reported rates of up to 40% [2]. Predictive models remain inconsistent and have not been routinely translated into clinical practice [3]. On the other hand, the loss of skeletal muscle mass (sarcopenia) was recently introduced as an objective parameter for outcome in hospitalized older patients [4]. Sarcopenia is usually verified through muscle function tests such as the gait speed test and the grip strength test [5,6]. However, measuring muscle function with the abovementioned techniques often cannot be performed in CSDH patients because of the frequently present disturbances of consciousness and/or hemiparesis. However, an alternative evaluation of muscle mass and function was recently shown through the association of radiologically measured temporal muscle thickness (TMT) and outcome in brain tumor patients [7,8,9]. Further, CT scans (computed tomography) are routinely performed on CSDH patients, and TMT measurement is easy and rapid to implement. We therefore investigated TMT as an alternative method to evaluate muscle mass and investigated its role as an objective parameter for CSDH outcome.

## 2. Materials and Methods

### 2.1. Patients and Data Collection

All patients admitted to the neurosurgical department of the authors’ institution between August 2017 and June 2020 with the diagnosis of a CSDH were included in the analysis. The inclusion criteria were: (1) chronic subdural hematoma diagnosed by CT or MRI scan and (2) patients aged 18 years and above. The patient characteristics and medical data were collected using the institutional electronic database. For this retrospective analysis, ethical approval was obtained from the Ethics Committee of the University Medicine Rostock, Germany (identification number: A 2021-0112). As a non-interventional monocentric study, patient consent was waived. The exclusion criteria were lack of radiological data or hospital discharge in less than 24 h after admission. The investigated medical record parameters included: age at admission, sex, GCS at admission, anticoagulation status, preexisting conditions, symptoms at admission, radiological parameters such as hematoma diameter and midline shift, clinical course, and status at discharge and at 3 months after operation.

### 2.2. Image Analysis 

**Figure 1:**
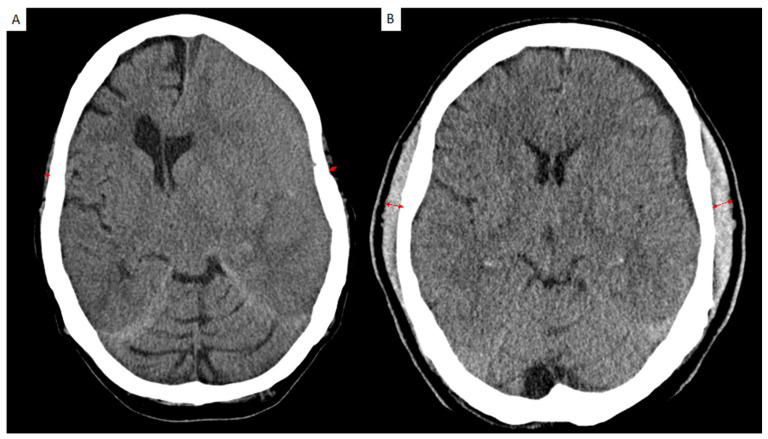
Representative case for the assessment of temporalis muscle thickness (TMT) on cranial CT scan shown in red arrows. (**A**) TMT measurement on axial CT scan image of a patient with low-volume TMT (bilateral mean TMT = 2.3 mm) and (**B**) a patient with a high-volume TMT on axial CT scan analysis (bilateral mean TMT = 8.1 mm).

Preoperative, postoperative, and follow-up CT scans were analyzed with PACS software Jivex^®^ v5.2 (VISUS Technology Transfer GmbH, Bochum, Germany). Image analysis was performed by two neurosurgeons (D.D. and S.Y.-W.) that were blinded to the patients’ medical data. A representative analysis is displayed in Figure 1. TMT was measured on the left and on the right side separately in each patient and each side was summed up and divided by two, resulting in a mean TMT per patient. The TMT side showed no statistical difference. High-volume TMT was defined as mean of 6–9 mm and low-volume TMT as 1–5 mm. 

### 2.3. Surgical Treatment

Unless there was a known history of cephalosporin allergy, all patients received a perioperative intravenous cefazolin (2 g) prophylaxis for evacuation. A closed subdural drainage system after evacuation was implanted in all cases. A postoperative CT scan was obtained prior to drain removal. Prophylactic low molecular weight heparin was started after 24 h in all patients. In cases with preoperative anticoagulation, the anticoagulant was re-administered not earlier than postoperative day 7. Recurrence was defined as the accumulation of chronic subdural fluid requiring re-operation. A second surgery performed during the same hospitalization was not considered as a recurrence. 

### 2.4. Study Design

The present analysis is a retrospective, single center study of patients with CSDH. The aim of the study was to observe patients’ TMT on preoperative CT scans and to correlate the TMT volume with patient outcomes.

### 2.5. Statistics

The data analysis was performed with IBM SPSS Statistics version 23.0 (SPSS Inc., IBM Corp, Armonk, NY, USA). For the patient characteristics, descriptive statistics were used. Fisher’s exact test was used for the comparison of categorical variables between the cohorts. For continuous parameters, the Wilcoxon/Mann–Whitney test was used. Bivariable and multi-variable logistic regression models were used to find correlations with TMT. The multivariable logistic regression analysis included variables with a *p*-value of less than or equal to 0.05 in the bivariable regression. A *p*-value < 0.05 was considered to determine statistical significance. To assess the impact of the variables, odds ratios (ORs) with 95% confidence intervals (CIs) were calculated. Results with *p* ≤ 0.05 were considered statistically relevant.

## 3. Results

### 3.1. Participant Characteristics

A total of 173 CSDH patients were analyzed. Two patients were excluded due to lack of radiological data, therefore a total of 171 CSDH patients were included in the final analysis. The mean TMT was 5 mm (IQR 3–9). The average age was 74.5 (IQR: 63–82) and 115 (67%) of the patients were male. The median GCS at admission was 15 (IQR: 14–15) and 96 patients (56%) received anticoagulation at admission. In terms of preexisting conditions, 112 patients presented with a history of hypertension (71%), 39 with atrial fibrillation (23%), 47 with diabetes mellitus (27%), 77 with coronary heart disease (45%), and 21 with dementia (12%). The symptoms at admission were headache in 59 cases (35%), confusion in 35 (20%), impaired consciousness in 40 (23%), nausea in 16 (9%), hemiparesis in 119 (70%), and seizure in 9 (5%). On initial axial CT scan, the maximal hematoma width was 18.4mm (IQR: 12–25) and the median width was 3.5mm (IQR: 4–6). The median midline shift was 6mm (IQR: 2–9). A total of five patients had postoperative seizures, six patients had early (<7 postoperative days) seizures (4%), four patients late (>7 postoperative days) seizures (3%), and one patient had status epilepticus (1%). Recurrence was seen in 56 patients (33%) and the modified Rankin scale at 3 months was 1.5 (IQR: 0.5–3) (Table 1). 

### 3.2. Characteristics and Admission Status in CSDH According to Temporal Muscle Thickness

In our uni- and multivariate analysis, male patients showed an association with low-volume TMT (59% of patients with low-volume TMT were male vs. 76% in the high-volume TMT cohort; *p* = 0.022). Furthermore, uni- and multivariate analysis showed a significant association between low-volume TMT and increased age (median age of 79 in the low volume TMT cohort vs. 70 years in the high-volume TMT cohort; *p* = 0.001). The GCS at admission as well as the anticoagulation status were not significantly associated with TMT volume (*p* = 1 for GCS and *p* = 0.089 for anticoagulation status). (Table 2)

### 3.3. Preexisting Conditions and Temporal Muscle Thickness in Patients with CSDH

In our analysis, atrial fibrillation, diabetes mellitus, and coronary heart disease showed no significant association with TMT volume (*p* = 1; *p* = 0.606 and *p* = 0.760, respectively) (Table 3). However, in uni- but not multivariate analysis, 78% of the patients with low-volume TMT had a history of hypertension vs. 51% of patients with high-volume TMT (*p* = 0.043 in univariate and *p* = 0.102 in multivariate analysis). Furthermore, in the univariate analysis, patients with low-volume TMT showed a history of dementia in 20% vs. 3% in patients with high-volume TMT (*p* = 0.001 for univariate). Multivariate analysis showed approximating significance with *p* = 0.05.

### 3.4. Symptoms at Admission and Temporal Muscle Thickness in Patients with CSDH

In the univariate analysis, headache as the leading symptom was significantly associated with low-volume TMT (26% in the low-volume TMT cohort vs. 35% in high-volume TMT, *p* = 0.023). Furthermore, confusion was present in 36% of patients with low-volume TMT vs. 15% in the high-volume cohort, which was statistically significant (*p* = 0.001). Nausea was present in 3% of the low-volume TMT vs. 16% in the high-volume TMT cohort, an association that is also statistically significant in univariate analysis (*p* = 0.004). Impaired consciousness, hemiparesis, and seizures were not significantly associated with patients’ TMT volume. Multivariate analysis only confirmed a significant association for nausea (*p* = 0.054). 

### 3.5. Association of Radiological Parameters and TMT in Patients with CSDH

The median hemorrhage diameter in patients with low-volume TMT was 20 mm (IQR 13–25) vs. 17 mm (IQR 11–25) in patients with high-volume TMT, which showed a statistical significance in the univariate (*p* = 0.019) and multivariate analysis (*p* = 0.012). On the other hand, the midline shift was not statistically associated with patients’ TMT volume (*p* = 1). 

### 3.6. Postoperative Seizures and Temporal Muscle Thickness in Patients with CSDH

Neither early nor postoperative seizures were statistically associated with patients’ temporal muscle thickness: *p* = 0.420 for early and *p* = 0.623 for late postoperative seizures. Status epilepticus was observed in only one patient among the analyzed cohort and was therefore not statistically significant (*p* = 1). 

### 3.7. Clinical Outcome and Temporal Muscle Thickness in Patients with CSDH

The GCS at discharge showed no significant association with patients’ TMT volume, with 15 in both groups (*p* = 1). However, mRS showed a significant association (*p* = 0.001) with a median mRS of 2 (IQR 1–3) in the low-volume TMT cohort vs. 1 in the high-volume TMT cohort (IQR: 0.75–2). This statistical significance was not observed in the multivariate analysis. Regarding the CSDH recurrence, no significant association was observed in the analyzed cohort: 27% in patients with low-volume TMT vs. 29% with high-volume TMT (*p* = 0.415). Furthermore, the 3-month follow-up confirmed better mRS status in the high-volume TMT cohort (1 vs. 2 in the low-volume cohort). This statistical significance could not be replicated in multivariate analysis.

## 4. Discussion

This study investigates the value of TMT volume on conventional preoperative CT scans preformed on patients with CSDH. The major finding is the significant association of low-volume TMT and increased hematoma volume as well as decreased outcome status at 3 months. The findings of this study suggest that TMT may represent an objective parameter with prognostic value, novel for CSDH patients. 

Frailty and sarcopenia are emerging as (substantially overlapping) parameters that are gaining recent scientific attention since both showed an association with a wide range of ageing outcomes and were shown to be predictive for mortality and morbidity in the elderly [10]. In the case of frailty, recent studies argued for the incorporation of frailty assessment into risk models for mortality and outcome in elderly patients with CSDH [11]. Sarcopenia, on the other hand, has been shown to be a negative prognostic factor for the outcome of brain tumor patients, although the underlying pathophysiology remains unclear. Among the discussed hypotheses were the association of TMT with general physical fitness, insufficient nutrition status, or inflammatory processes [12]. A possible crossover of this finding to CSDH patients is unexamined at this time.

In terms of TMT as a general physical fitness parameter, several studies confirmed the reliability of sarcopenia as a useful assessment instrument in older trauma patients. Tanabe et al. could show an association of sarcopenia and increased 1-year mortality in older trauma patients [13]. However, in their analysis, a different muscle group (masseter) as well as distinct measuring techniques were used. Our results are in line with the available literature, but extend the applicability to the CSDH disease pattern.

Patients’ nutritional status is certainly of significant importance for postoperative outcome in CSDH patients. Scaretti et al. recently showed that reduced nutritional status analyzed via Mini Nutritional Assessment (MNA) in 178 CSDH patients was a strong predictor of poor clinical outcome after hematoma evacuation [14]. Our analysis confirms the previously mentioned negative association between reduced nutritional status and poor outcome as our cohort with a low-volume TMT showed similarly poor clinical outcome. Furthermore, in their multivariate analysis of SAH patients, Katsuki et al. confirmed TMT as a prognostic factor in older patients. Similar results were seen in ischemic stroke patients [15,16,17]. Our findings add CSDH as a common neurosurgical condition to the list of previous publications on the prognostic value of TMT in intracranial hematoma patients.

With regard to the possible interaction between inflammatory processes and TMT volume, several studies highlighted the possible component of inflammation in the formation and maintenance of CSDH. Local inflammatory cells including neutrophils, lymphocytes, monocytes, and eosinophils have been observed on the outer CSDH membrane [18]. Inflammation regulates muscle protein metabolism and is known to be associated with a chronic state of slightly increased plasma levels of pro-inflammatory mediators, such as tumor necrosis factor α (TNFα), interleukin 6 (IL-6), and C-reactive protein (CRP) [19]. Whether sarcopenia as a possible consequence of chronic inflammation in CSDH has its rationale and influences hematoma membrane formation and maintenance remains speculative at this time since our analysis did not show a significant impact on recurrence rates. 

Another interesting finding of our study is the significant association of TMT and dementia. The loss of muscle mass is known to be associated with brain atrophy in Alzheimer’s disease (AD), and skeletal muscle mass is proportionately reduced in patients with dementia compared to those with mild cognitive impairment [20]. Our analysis confirms this aspect, as patients with reduced TMT volume (hence, reduced muscle mass) had significantly more often dementia as a subsidiary diagnosis.

Furthermore, several studies could show that protocols for automatic geriatrician consultation in trauma patients 70 years and older resulted in improved advanced care planning and increased multidisciplinary care. In the ICU, advanced care planning reduces readmission and length of stay [21]. In summary, the major possible benefit of implementing TMT values in clinical routine should be the early identification of vulnerable CSDH patients that are at high risk of poor outcome.

## 5. Limitations

Although our analysis shows the value of TMT in a sizable cohort of CSDH patients, our study faces some limitations. Firstly, after stratification by hematoma volume, our cohort showed a significant age difference, which could influence outcome. However, previous studies could not show a significant association between patients’ age and TMT volume [22]. Further, we performed multivariable statistical approaches to consider this effect. The statistically significant overrepresentation of the male sex in CSDH is a known phenomenon that should therefore not present a clinically relevant bias [23]. Secondly, the retrospective analysis of TMT prohibited the evaluation of anatomical–functional relationships. Furthermore, patients’ blood type was previously described as a significant risk factor for rebleeding in acute SDH and SAH patients [24,25,26]. Due to the lack of patients’ blood type, we could not include this parameter in our analysis, but stress the necessity for future studies. Furthermore, postoperative pneumocephalus following CSDH evacuation has been found to be a risk factor for re-accumulation, and we did not analyze the incidence of pneumocephalus. Future studies should investigate its role in CSDH recurrence. Even though recent studies have shown a direct correlation between TMT and sarcopenia, a generalization in terms of sarcopenia remains to be proven in future studies [27,28]. As this is a retrospective observational study, confounding, selection bias, and uncontrolled statistical error risk cannot be excluded. Hence, further prospective randomized trials with large cohorts are necessary to validate our findings. 

## 6. Conclusions

The results described in this study could pave the way for the implementation of TMT measurement for the assessment of sarcopenia in CSDH and therefore identify vulnerable patients with the opportunity of postoperative follow-up optimization.

## Figures and Tables

**Table 1:** Demographics, management, and surgical data. IQR: Inter quartile range; GCS: Glasgow Coma Score; mRS: Modified rankin scale.

Patient Characteristics	(*n* = 171)
Sex	
male, *n* (%)	115 (67)
Age, median (IQR)	74.5 (63–82)
GCS at admission, median (IQR)	15 (14–15)
Anticoagulation, *n* (%)	96 (56)
Preexisting conditions	
Hypertension, *n* (%)	122 (71)
Atrial fibrillation, *n* (%)	39 (23)
Diabetes mellitus, *n* (%)	47 (27)
Coronary heart disease, *n* (%)	77 (45)
Dementia, *n* (%)	21 (12)
Symptoms at admission	
Headache, *n* (%)	59 (35)
Confusion, *n* (%)	35 (20)
Impaired consciousness, *n* (%)	40 (23)
Nausea, *n* (%)	16 (9)
Hemiparesis, *n* (%)	119 (70)
Seizure, *n* (%)	9 (5)
Radiological parameters	
Hematoma median, mm (IQR)	18.4 (12–25)
Midline-shift, median, mm (IQR)	6 (2–9)
Postoperative seizures	
Early seizure (<7 d), *n* (%)	6 (4)
Late seizure (>7 d), *n* (%)	4 (3)
Status epilepticus, *n* (%)	1 (1)
Status at discharge	
GCS at discharge, median (IQR)	15 (15–15)
mRS at discharge, median (IQR)	2 (1–3)
Recurrence, *n* (%)	56 (33)
Outcome	
mRS3 months, median (IQR)	1.5 (0.5–3)

**Table 2:** Univariate analysis of juxtaposed characteristics according to TMT volume in CSDH. Abbreviations: OR: odds ratio, IQR: interquartile range, GCS: Glasgow Coma Scale, CRP: C-reactive protein.

Patient Characteristics (*n* = 171)	Mean TMT		Univariate		
	Low Volume *n* = 91	High Volume *n* = 80	OR	95% CI	*p*-Value
Sex					
male, *n* (%)	54 (59.3)	61 (76.3)	0.5	0.23–0.88	0.022
Age, median (IQR)	79 (71–84)	70 (56–79)	-	4.58–13.42	0.000
GCS at admission, median (IQR)	15 (14–15)	15 (14–15)	-	−0.44–0.44	1
Anticoagulation, *n* (%)	57 (62.6)	39 (48.8)	1.8	0.96–3.25	0.089
Preexisting conditions					
Hypertension, *n* (%)	71 (78)	51 (63.8)	2	1.03–4.00	0.043
Atrial fibrillation, *n* (%)	21 (23.1)	18 (22.5)	1	0.51–2.12	1
Diabetes mellitus, *n* (%)	27 (29.7)	20 (25.0)	1.3	0.64–2.50	0.606
Coronary heart disease, *n* (%)	42 (46.2)	35 (43.8)	1.1	0.60–2.02	0.760
Dementia, *n* (%)	18 (19.8)	3 (3.8)	6.3	1.79–22.39	0.001
Symptoms at admission					
Headache, *n* (%)	24 (26.4)	35 (43.8)	0.5	0.24–0.88	0.023
Confusion, *n* (%)	33 (36.3)	12 (15.0)	3.2	1.53–6.81	0.001
Impaired consciousness, *n* (%)	20 (22.0)	20 (25.0)	0.9	0.42–1.72	0.718
Nausea, *n* (%)	3 (3.3)	13 (16.3)	0.2	0.10–0.64	0.004
Hemiparesis, *n* (%)	39 (42.9)	40 (50.0)	0.8	0.41–1.37	0.361
Seizure, *n* (%)	5 (5.5)	4 (5.0)	1.1	0.29–4.26	1
Radiological parameters					
Hematoma median, mm (IQR)	20 (13–25)	17 (11–25)	1.5	0.49–5.1	0.019
Midline-shift, median, mm (IQR)	6 (2–8)	6 (2–11)	-	−1.44–1.44	1
Postoperative seizures					
Early seizure (<7 d), *n* (%)	2 (2.2)	4 (5.0)	0.4	0.08–40	0.420
Late seizure (>7 d), *n* (%)	3 (3.3)	1 (1.3)	2.7	0.27–26.43	0.623
Status epilepticus, *n* (%)	1 (1.1)	0 (0)	-	-	1
Status at discharge					
GCS at discharge, median (IQR)	15 (15–15)	15 (15–15)	-	−0.13–0.13	1
mRS at discharge, median (IQR)	2 (1–3)	1 (0.75–2)	-	0.58–1.42	0.001
Recurrence, *n* (%)	27 (29.7)	29 (36.3)	0.7	0.39–1.41	0.415
Outcome					
mRS3 months, median (IQR)	2 (1–4)	1 (0–2)	-	0.36–1.63	0.002

**Table 3:** Uni- and multivariate analysis of juxtaposed characteristics according to TMT volume in CSDH. Abbreviations: OR: odds ratio, IQR: interquartile range, GCS: Glasgow Coma Scale, CRP: C-reactive protein.

Patient Characteristics (*n* = 171)	Mean TMT		Univariate			Multivariate		
	1–5 mm *N* = 91	6–9 mm *N* = 80	OR	95% CI	*p*-Value	OR	95% CI	*p*-Value
Sex								
Male, *n* (%)	54 (59.3)	61 (76.3)	0.5	0.23–0.88	0.022	2.8	1.31–6.05	0.008
Age, median (IQR)	79 (71–84)	70 (56–79)	-	4.58–13.42	0.001			
Preexisting conditions								
Hypertension, *n* (%)	71 (78.0)	51 (63.8)	2	1.03–4.00	0.043	0.5	0.25–1.14	0.102
Dementia, *n* (%)	18 (19.8)	3 (3.8)	6.3	1.79–22.39	0.001	0.3	0.07–1.0	0.050
Symptoms at admission								
Headache, *n* (%)	24 (26.4)	35 (43.8)	0.5	0.24–0.88	0.023	1.7	0.78–3.58	0.183
Confusion, *n* (%)	33 (36.3)	12 (15.0)	3.2	1.53–6.81	0.001	0.5	0.21–1.21	0.124
Nausea, *n* (%)	3 (3.3)	13 (16.3)	0.2	0.10–0.64	0.004	4.1	1.0–17.43	0.054
Radiological parameters								
Hematoma median, mm (IQR)	20 (13–25)	17 (11–25)	1.5	0.49–5.1	0.019	8.1	0.26–0.61	0.012
Outcome								
mRS3 months, median (IQR)	2 (1–4)	1 (0–2)	-	0.36–1.63	0.002	0.2	0.58–1.36	0.613

## Data Availability

The data presented in this study are available on request from the corresponding author.
